# Brown Macroalgae as Valuable Food Ingredients

**DOI:** 10.3390/antiox8090365

**Published:** 2019-09-02

**Authors:** Nuno C. Afonso, Marcelo D. Catarino, Artur M. S. Silva, Susana M. Cardoso

**Affiliations:** QOPNA & LAQV-REQUIMTE, Department of Chemistry, University of Aveiro, 3810-193 Aveiro, Portugal

**Keywords:** *Phaeophyceae*, food fortification, algae, fibres, phlorotannins, fucoxanthin, minerals, iodine, nutrition, health-benefits, functional food

## Abstract

Due to the balanced nutritional value and abundance of bioactive compounds, seaweeds represent great candidates to be used as health-promoting ingredients by the food industry. In this field, Phaeophyta, i.e., brown macroalgae, have been receiving great attention particularly due to their abundance in complex polysaccharides, phlorotannins, fucoxanthin and iodine. In the past decade, brown algae and their extracts have been extensively studied, aiming at the development of well-accepted products with the simultaneous enhancement of nutritional value and/or shelf-life. However, the reports aiming at their bioactivity in in vivo models are still scarce and need additional exploration. Therefore, this manuscript revises the relevant literature data regarding the development of Phaeophyta-enriched food products, namely those focused on species considered as safe for human consumption in Europe. Hopefully, this will create awareness to the need of further studies in order to determine how those benefits can translate to human beings.

## 1. Introduction

Marine macroalgae i.e., seaweeds, have been well recognised for centuries by their importance in the diet of many Far Eastern countries, such as Japan and Korea [1,2]. They are nutritionally very wealthy, being claimed as a great source of complex polysaccharides, minerals, proteins and vitamins, as well as of several phycochemicals [3,4]. Actually, a regular seafood consumption, in which seaweeds are included, has been associated with a myriad of health benefits and a longer life expectancy [5,6] and these combined facts are leading to an increased interest in the manufacture and consumption of high-value macroalgae-derived products in Western cultures. Their consumption is also in line with the increasing awareness of consumers’ perceptions towards organic products and of environmentally sustainable products. As a result, according to the Seafood Source report, the global seaweed market is expected to grow to USD 22.1 billion by 2024 [7].

Nowadays, amongst all three types of macroalgae (green, red and brown), brown algae are the most consumed species (66.5%), followed by red (33%) and green (5%) algae [8]. *Phaeophyceae* possess a high content of diverse phycochemicals and have been repeatedly claimed to exert important therapeutic properties, which turn them into great candidates to be used as bioactive agents in many industries, including the functional food market [9,10,11].

Europe has been recently highlighted as one of the most innovative regions regarding the use of seaweeds as a food ingredient with new products emerging on the European market increasing at exponential rates [12]. In fact, according to the Seafood Source report, the new products containing this new ingredient launched on the European market increased by 147% between 2011 and 2015, making Europe the most innovative region globally after Asia [13]. In this region, algae are considered as novel foods and a limited number of brown macroalgae species are considered to be safe for human consumption, namely *Fucus vesiculosus, Fucus serratus, Himanthalia elongata*, *Undaria pinnatifida*, *Ascophyllum nodosum*, *Laminaria digitata, Laminaria saccharina, Laminaria japonica* and *Alaria esculenta* [14]. The present manuscript reviews the current knowledge on the incorporation of these brown seaweed species and brown seaweed-derived high-value products in common food products.

## 2. Chemical Particularities of Brown Macroalgae

The health-claims of *Phaeophyceae* are mainly associated with their abundance in specific nutrients and phycochemicals, particularly fibres, phlorotannins, fucoxanthin and minerals. However, their levels are greatly variable according to distinct factors, including the algae genera and species, maturity and the environmental conditions, i.e., the variations to which the natural habitat of algae might be subjected, namely season, temperature, salinity, oceanic currents, waves or even depth of immersion, as well as post-harvesting storage and processing conditions [9,10,15,16,17,18]. As such, this section describes their main structural characteristics as well as some of their most relevant bioactivities, highlighting their overall abundance in the targeted macroalgae of this review.

### 2.1. Polysaccharides

Brown macroalgae are known to produce different types of polysaccharides and/or fibres which, despite their variability, represent major components that can reach up to 70% of their dried weight (DW) [19]. In fact, previous reported data set the polysaccharide contents of relevant species, namely *L. japonica*, *F. vesiculosus*, *A. nodosum*, *Saccharina longicruis*, *U. pinnatifida* and *Sargassum vulgare* at 37.5%, 65.7%, 69.6%, 57.8%, 35.2% and 67.8% DW, respectively [20,21,22,23]. Amongst them, alginates, fucoidans and laminarins are the most representative ones.

**Figure 1:**
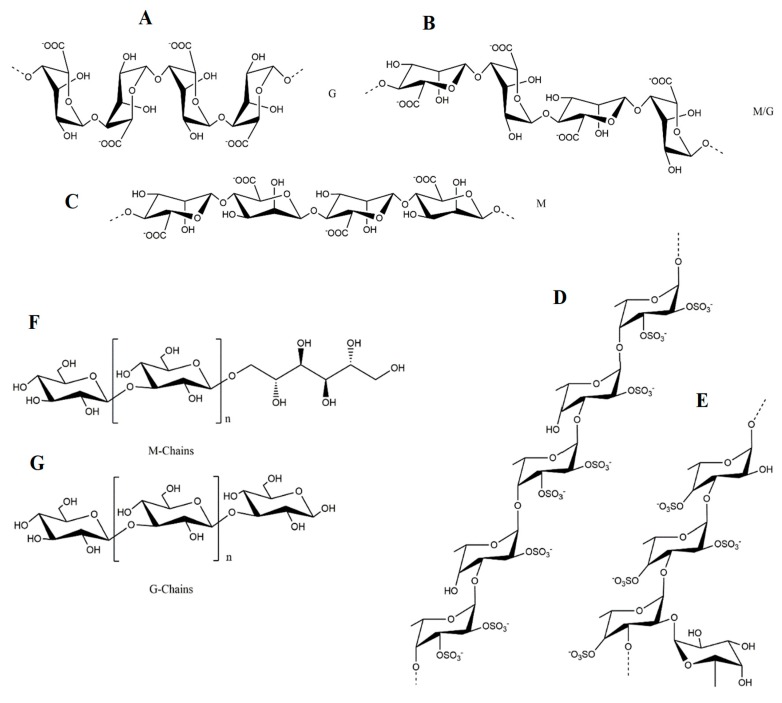
The structure of representative polysaccharides found in brown algae: (**A**–**C**) alginic acids; (**D**–**E**) fucoidans from *A. nodusum/F. vesiculosus* and *S. latissima*, respectively; (**F**–**G**) laminarins M and G chains.

Alginic acids or alginates, i.e., the salts of alginic acid, are the main polysaccharides in brown seaweeds [24], reaching up to 16.9% DW in *S. vulgare*, 20% DW in *S. longicruris*, 24% DW in *A. nodosum*, 32% DW in *Sargassum carpophyllum*, 40% DW in *Laminaria hyperborean* [25], 41% in *Sargassum siliquosum* and even to 59% DW in *F. vesiculosus* [26]. Within the cell wall, these polysaccharides are known to be partially responsible for the seaweed’s flexibility [3] and therefore, expectedly, brown seaweeds grown under turbulent conditions usually have superior alginate contents than those of calm waters. In terms of structure, alginic acids or their corresponding extracted salts consist of α-l-guluronic acid (G) and β-d-mannuronic acid (M) (1→4)-linked residues arranged either in heteropolymeric (MG) and/or homopolymeric (M or G) blocks (Figure 1A–C). Regardless, the variations caused by diverse factors (e.g., algae species, seasonability, parts of the algae) are expected [16]. Noteworthy, alginates are considered one of the most important food colloids, with many applications in several industries such as foods, paper, pharmaceutical or cosmetics [27]. In fact, G-blocks in the presence of ions, such as Ca^2+^ form is the so-called egg-box, thus granting stiffness to the overall structure and conferring gel-forming properties to these polysaccharides [28]. Therefore, they are usually used as thickeners, gels, emulsifiers and stabilizers in order to improve quality parameters, especially in food grade products [29]. In addition to their wide applications, more recently, dietary alginates are being associated with positive health benefits in the gastrointestinal tract and appetite regulation, as well as antihypertensive and anti-diabetic effects [30]. Alginates are also considered great prebiotics as they were demonstrated to significantly promote the growth of several bacteria, including *Bifidobacterium bifidum*, *Bifidobacterium longum* and *Lactobacilli*, alongside with the increase of acetic acid, propionic acid and several short chain fatty acid metabolites, while decreasing deleterious metabolites, including faecal sulphide, phenol, *p*-cresol, indole, ammonia and skatole [31].

Fucoidans i.e., metabolites belonging to the fucans family, also have a structural role in brown algae, mostly preventing dehydration [3]. Their reported content in *Phaeophytae* is variable, ranging from approximately 6–8% DW in *L. japonica*, 3.2–16% DW in *U. pinnatifida*, and 3.4–25.7% DW in *F. vesiculosus* [10,32]. These polysaccharides are mainly composed of fucose and sulphate, although the presence of other types of monosaccharides (glucose, galactose, mannose, xylose and uronic acids), acetyl groups and proteins also occur [33]. Despite being molecules with high structural diversity, the representative backbone of fucoidans consists of (1→3)- and (1→4)-linked α-l-fucopyranose residues, and these polysaccharides are commonly divided in two types, the first being characterized by long chains of (1→3)-linked α-l-fucopyranose residues (mainly present in *L. saccharina*, *L. digitata*, *C. okamuranus*, and *Chorda filum*) and the second consisting of alternating (1→3)- and (1→4)-linked α-l-fucopyranose residues (characteristic from *A. nodosum* and *Fucus* spp.) (Figure 1D,E) [24,34].

Over the last years, extensive biological activities (e.g., antitumor, antioxidant, anticoagulant, antithrombotic, immunoregulatory, antiviral, anti-inflammatory among others) have been demonstrated with promising preclinical results, as recently reviewed [35]. As an example of in vivo studies, the effectiveness of a *F. vesiculosus* fucoidan injection towards oxidative stress in hyperoxaluric rats was demonstrated by Veena et al. [36] to be mediated by the stimulation of antioxidant enzymes, such as superoxide dismutase (SOD), catalase (CAT) and glutathione peroxidase (GPx). Moreover, Huang et al. [37] reported that the ingestion of fucoidans isolated from *L. japonica* reduced the serum levels of total cholesterol, triglycerides, and low-density lipoprotein cholesterol in hyperlipidaemic rats, while increasing the enzymatic activity of lipoprotein lipase, hepatic lipase and lecithin cholesterol acyltransferase. In addition, their relevance in obesity and/or diabetes was also highlighted, in particular, by Xan et al. [38], who reported *F. vesiculosus* fucoidans’ ability to inhibit α-glucosidase in vitro and to decrease the fasting blood glucose and glycosylated haemoglobin levels of db/db mice, as well as by Kim et al. [39], when administrating *U. pinnatifida* fucoidans to the same animal model. Although there is limited evidence to implicate a role of fucoidans in the gut microbiota, some works reported that fucoidans from different brown algae species greatly contributed for the increase in the growth of *Bifidobacterium*, *Lactobacillus* and *Ruminococcaceae,* either in mice or human faecal samples [31].

Laminarins, also named laminarans or leucosins, on the other hand, belong to the glucan family and serve as reserve metabolites in brown algae [40]. These are commonly found in the fronds of *Laminaria* and *Saccharina* macroalgae and, to a lesser extent, in *Ascophyllum*, *Fucus* and *Undaria* species [41]. In general, they are relatively small polysaccharides composed of β-(1→3)-linked glucose monomers, containing large amounts of sugars and a low fraction of uronic acids (Figure 1F,G) [42]. Depending on the type of sugar at the reducing end, they are classified in two distinct types, specifically the M chains, which have a terminal 1-O-substituted d-mannitol, and the G chains, possessing a terminal glucose [16]. The content of these polysaccharides is also season-dependent, since seaweeds show no production or very less amounts in the winter and maximum production during summer and autumn [23]. As previously stated, *Laminariales* are known to produce high amounts of laminarins, with contents reaching up to 35% DW, particularly in *L. saccharina* and *L. digitata* [40]. Other reported values of laminarins content comprise those of *A. esculenta*, *U. pinnatifida*, *A. nodosum* and *F. serratus* (11.1%, 3%, 4.5% and up to 19% DW, respectively) [22,23,24,40].

The bioactivities of laminarins are scarcely exploited, but still they are considered as fibres and therefore can be partially or totally fermented by the endogenous intestinal microflora. This was demonstrated by Devillé et al. [43], when comparing the results from in vitro digestibility tests, where no hydrolysis of this fibre occurred, to those of in vivo tests, for which no traces of laminarin were detected in the faeces of fed Winstar rats after ingestion.

It should be noted that oligosaccharides from brown macroalgae polysaccharides may also exhibit interesting bioactivities, which can differ from those of the original polysaccharides. In this topic, alginate oligosaccharides have been claimed to possess radical scavenging activities with the great potential for application in the food industry [44], and even promising effects on neuro-inflammation, promoting microglial phagocytosis. This could be of great relevance for their application as a nutraceutical agent for neurodegenerative diseases, such as Alzheimer’s disease [45]. In turn, in vivo experiments on renovascular hypertensive rats revealed that fucoidan oligosaccharides exhibited anti-hypertensive effects comparable to those of captopril, i.e., an approved drug used for the treatment of hypertension [46].

### 2.2. Phlorotannins

Phlorotannins are phenolic compounds almost exclusive to *Phaeophytae* and also represent their main phenolic pool. In brown seaweeds, they are associated with a myriad of functions, ranging from structural cell wall components, to biosyinthetic percursors and defensive mediators against natural enemies, acting as herbivore deterrents, inhibitors of digestion and agents against bacteria [11]. Phlorotannins are known to accumulate mostly in physodes (i.e., specialized membrane-bound vesicles of the cell cytoplasm), with levels that might represent up to 25% of seaweed’s DW, despite variations which occur depending on distinct factors [47]. For example, the higher levels of phlorotannins in *Fucus* spp. are associated with high salinity waters and solar exposure during summer [10].

**Figure 2:**
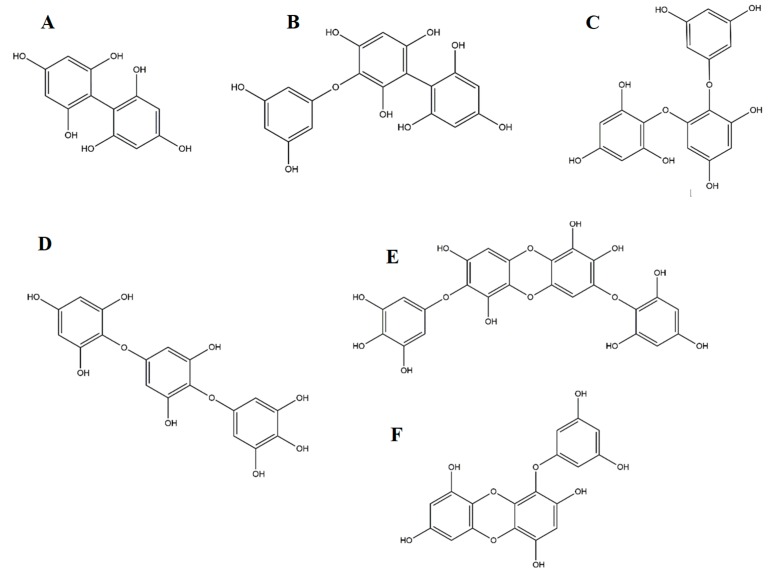
Some representative phlorotannins from brown seaweeds: (**A**) Fucol; (**B**) Fucophlorethol; (**C**) Phlorethol; (**D**) Fuhalol; (**E**) Carmalol; (**F**) Eckol.

Being part of the tannins group, phlorotannins present a polymeric structure derived from several phloroglucinol (1,3,5-trihydroxybenzene) units and possess a high number of hydroxy groups, thus conferring them solubility in water [48]. Depending on the linkage between phloroglucinol monomer units, a wide range of compounds with different molecular weights can be obtained [49], which overall, are divided in four categories for each type of linkage: Fuhalols and phlorethols based on ether linkage, fucols based on C-C linkage, fucophlorethols for a combination of the previous ones, and, finally, eckols and carmalols, based on dibenzodioxin linkage (Figure 2).

Phenolic extracts from brown seaweeds have been demonstrated to exhibit various biological activities, including antioxidant, antidiabetic, anti-inflammatory and others [11,50,51]. In this regard, O’Sullivan et al. [52] observed the augment of glutathione levels in Caco-2 cell models when incubated with *A. nodosum*, *F. vesiculosus* and *F. serratus* phlorotannins extracts, while also highlighting the protective effects of the latter on the same model pretreated with H_2_O_2_. In vivo experiments have even demonstrated that the oral administration of 200 mg/kg/day of *F. vesiculosus* polyhenol-rich extracts over 4 weeks to Sprague-Dawley rats could increase the blood plasma reducing power, paraoxonase/arylesterase 1 (PON-1) activity and O_2_^•−^ scavenging activity by 29%, 33% and 25%, respectively [53]. Likewise, the antidiabetic properties of *A. nodosum* and *F. vesiculosus* phenolic-rich extract were observed in vivo as the postprandial blood glucose levels and insulin peak decreased 90% and 40%, respectively, on rats under hyperglycemic diets supplemented with 7.5 mg/kg compared to the unsupplemented group [54]. In fact, the ingestion of 500 mg of this mixture containing *A. nodosum* and *F. vesiculosus* 30 min prior to the consumption of carbohydrates was shown to reduce the insulin incremental area of the curve and an increase in insulin sensitivity in a human clinical trial [55]. Human trials have also been carried out to evaluate the potential antiobesity effect of polyphenolic-rich extracts of *A. nodosum* (100 mg/day for 8 weeks). Although the treatment did not exhibit any significant benefits (no significant changes in C-reactive protein, antioxidant status or inflammatory cytokines), with the exception of a modest decrease of the DNA damage in the obese group, several phlorotannin metabolites were detected in the subjects plasma and urine, indicating that these compounds are metabolised and absorbed into the systemic circulation [56]. These observations are in line with those reported by Corona et al. [57] who also described the appearance of phlorotannin metabolites in urine and plasma collected from humans after consuming a capsule of *A. nodosum* extract containing about 100 mg of polyphenols.

### 2.3. Fucoxanthin

**Figure 3:**
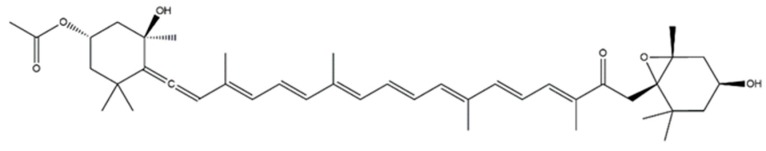
Structure of fucoxanthin.

In opposition to red and green macroalgae, *Phaophytae* are characterized by the presence of the carotenoid fucoxanthin, which is responsible for their specific coloration. Fucoxanthin is a xanthophyll belonging to the tetraterpenoid family with a structure consisting of an unusual allenic bond and a 5,6-monoepoxide in its molecule (Figure 3). The content of this pigment is highly variable amongst different species, as well as dependent on extrinsic factors, with a large range being even described within the same species. The reported levels comprise in 171 mg/kg (*Fucus spiralis*), 224 mg/kg (*Fucus distichus*), 364 mg/kg (*Fucus evanescens*), 172–660 mg/kg (*A. nodosum*), 178–468 mg/kg (*Laminaria* spp.) [41,58].

Recently, this xanthophyll has earned particular attention mainly because of its promising effects in terms of antidiabetic, anti-obesity and antioxidant activities [59,60], with claims being supported by in vivo studies. For instance, the administration of *U. pinnatifida* lipids rich in fucoxanthin to male diabetic mice were associated with insulin resistance amelioration and the reduction of blood glucose levels [61]. Moreover, fucoxanthin isolated from the same macraolgae species was also shown to inhibit the differentiation of 3T3-L1 preadipocytes into adipocytes by down-regulating peroxisome proliferator-activated receptor gamma (PPARγ) [62]. Furthermore, a diet based on *U. pinnatifida* fucoxanthin was capable of inducing uncoupling protein 1 (UCP1) expression in white adipose tissue (WAT) of obese mice. When added as a supplement to rats fed with a high-fat diet, it prompted a decrement of the mRNA expression of significant enzymes associated with lipid metabolism, such as fatty acid synthase, acyl-CoA cholesterol acyltransferase, hepatic acetyl-CoA carboxylase, glucose-6-phosphate dehydrogenase, hydroxy-3-methylglutaryl coenzyme A and SREBP-1C [63,64].

### 2.4. Minerals

Due to their structural and physiological features, brown macroalgae are recognized for their superior ability to accumulate minerals. Although the content of minerals like calcium, magnesium, phosphorus, potassium, sodium and iron is usually high within the macroalgae matrix, one of the standout aspects, comparatively to plants in general, are both their low Na/K ratios and high iodine levels [4]. In fact, it is well accepeted that low Na/K ratios are an important aspect for good maintenance of cardiovascular health [65]. Therefore, according to the World Health Organization (WHO), the recommended value for this should be close to one, so consumption of food products with this proportion or below should be considered for healthy cardiovascular purposes [66]. In fact, several studies point to a Na/K ratio ranging between 0.3 and 1.5 in brown seaweeds, with particular interest for *Laminaria* spp. (0.3–0.4) from Spain origins, wich are significantly lower than diverse food products, such as cheddar cheese (8.7), olives (43.6), and sausages (4.9) [4,67,68,69,70]. Additionally, *Phaeophyceae* seaweeds, due to the rich composition in alginates and sulphated polysaccharides coupled with the presence of haloperoxidases in the cell walls, allow the accumulation of iodine to more than 30,000 times over its concentration in the surrounding environment which is even higher than any edible plant [71]. The major contents of iodine were documented for *L. digitata*, *A. nodosum*, *H. elogata* and *U. pinnatifida* exhibiting concentrations of 70, 18.2, 10.7 and 3.9 mg/100 g wet weight, respectively [72]. Moreover, other studies also highlight the particular affinity of Laminarales to accumulate iodine, particularly *L. digitata,* in which values are known to reach 9014 and 8122 mg/kg DW, in spring and autumn, respectively [23].

## 3. Use of Brown Macroalgae as Food Ingredient

Being considered as a rich and balanced source of nutrients and bioactive compounds, consumers and food industries have a growing interest to introduce macroalgae, including *Phaeophytae*, into the dietary habits of the western countries, with new products already being launched in the markets at high rates in Europe. The usage of brown species as food ingredients has, however, to overcome huge challenges, that go from the guarantee of enough biomass to sustain the market development, to the gain of consistent knowledge of their physicochemical features, as well as understanding the extension of their impact when used as ingredients in foods. This section highlights some of the developed foods in the field of seaweed-fortified products, categorized by the respective incorporated algae species, considering the authorized seaweeds for human consumption in France/Europe [14], and finalising with the influence this incorporation has on the foods’ chemical, functional and structural behaviour.

### 3.1. Fucus vesiculosus

*F. vesiculosus* has found application as a functional ingredient in many different food matrices, mostly as a source of phlorotannins and antioxidant compounds, aiming to prevent food spoilage resultant from oxidative deterioration (Table 1). Fish and fish-derived products are one of the main matrices where several studies with this seaweed have been conducted. In this context, Dellarosa et al. [73] reported that neither aqueous nor 80% ethanol extracts from *F. vesiculosus* had significant effects on the lipid oxidation of fish cakes enriched with omega-3 polyunsaturated fatty acids, throughout a 28-days refrigerate storage. Nevertheless, the authors showed that no off flavour was detected in any samples tested, with low scores of rancid odour and flavour being registered in the sensory analysis. On the other hand, some studies conducted on cod fish muscle and/or protein indicated that the incorporation of *F. vesiculosus* extracts could indeed prevent the lipid peroxidation events and even improve some of their sensorial aspects. In fact, the effects of the incorporation of 1% and 2% of the antioxidant dietary fibre extracted from *F. vesiculosus* into minced horse mackerel revealed a significant reduction of the fish mince lipid oxidation throughout the 5 months of storage at –20 °C. These factors reduced the total drip after thawing and cooking the horse mackerel mince up to 3 months of frozen storage, a fact that could be due to the water holding capacities of the fibre. Furthermore, although the addition of 2% (but not 1%) of antioxidant dietary fibre caused changes in the fish mince flavour compared to the control, these were actually considered positive by the sensory panellists [74].

In a different approach, Wang et al. observed that some oligomeric phlorotannin sub-fractions obtained by Sephadex LH-20 chromatography from an 80% ethanol extract of *F. vesiculosus* were able to completely inhibit the haemoglobin-catalysed lipid oxidation in both washed cod muscle and cod protein isolates systems, during an 8-day period of ice storage. Moreover, with a concentration of 300 mg/kg, the effectiveness of these phlorotannins sub-fractions were comparable to that of 100 mg/kg propyl gallate, i.e., a highly effective synthetic antioxidant in muscle foods, thus evidencing the great potential of oligomeric phlorotannins to be exploited as natural antioxidants in fish and fish-derived products [75]. Similar results were further reported by Jónsdóttir et al. [76], who observed an inhibition of the lipid oxidation in haemoglobin-fortified washed cod mince system after incorporating 300 mg phloroglucinol equivalents/kg of an ethyl acetate fraction obtained from an 80% ethanol extract of *F. vesiculosus*. Other authors also demonstrated that the incorporation of a *F. vesiculosus* phlorotannin-rich fraction (obtained with 80% ethanol and further purified with ethyl acetate) into cod protein hydrolysates, not only prevented the lipid oxidation reactions during storage, but also increased their final antioxidant activity [77,78] and could even improve the bitter, soap, fish oil and rancidity taste of the final protein hydrolysates [77].

The fortification of food matrices with fish oils rich in n-3 long chain polyunsaturated fatty acids has been in high demand during recent years due to increasing consumer awareness of the beneficial effects of docosahexaenoic and eicosapentaenoic acids (DHA and EPA, respectively). However, this usually decreases the foods’ oxidative stability, leading to the development of undesirable off-flavours and consequent shelf-life reduction [88]. In this field, *F. vesiculosus* extracts were found to be highly promising. According to Karadağ et al., [79] the introduction of 0.5 and 1 g/100 g of both *F. vesiculosus* ethanol and acetone extracts into fish oil-enriched granola bars effectively improved their lipid stability, contributing to an increase of the foods’ phenolic content, radical scavenging activity, interfacial affinity of phenolics and eventual regeneration of tocopherol, which consequently cause the reduction of the iron-lipid interactions as well as the lipid oxidation during the storage period. These results agree with previous data demonstrating that addition of both ethanol and acetone *F. vesiculosus* extracts to granola bars enriched with multi-layered fish oil emulsion contributed to the reduction of the formation of primary and secondary oxidation products over the period of storage at 20 °C [80]. Enhancement of lipid stability was also described in two other fish oil-fortified food matrices, namely mayonnaise and milk, after incorporation of 1.0–2.0 g/100 g of an ethyl acetate fraction, obtained from *F. vesiculosus* 80% ethanol extract (rich in phenolics and carotenoids) [81], as well as in fish oil-fortified mayonnaise added with 1.5–2.0 g/kg of both acetone and ethanol extracts of this seaweed species [82]. Interestingly, in the particular case of fish oil-fortified mayonnaise, Hermund et al. [81] found that, despite its lower content of phenolics and carotenoids, *F. vesiculosus* water extracts, at high concentrations, could prevent the peroxides formation more effectively than the ethyl acetate fraction, much likely due to its higher metal chelating capacity resultant from the presence of polysaccharides or other highly polar compounds with strong metal chelating capacities. This outcome was, however, refuted in a latter study that reported an increased peroxide formation in fish oil-enriched mayonnaise also incorporated with *F. vesiculosus* water extracts [82]. The disparity found between these two works might be related to the differences in the trace metal contents of the aqueous extracts performed in each study since the former had much lower iron content than the latter, which might be responsible for the induction of lipid oxidation in the food matrix.

Recently, the fortification of canola oil with 500 ppm of *F. vesiculosus* water extract was reported to reduce approximately 70% of the peroxides formation and 50% of the thiobarbituric acid reactive substances (TBARS) value compared to the control samples, both under accelerated storage conditions (60 °C). This confirms that this extract may in fact hold the potential to be exploited as a food antioxidant agent. Indeed, under similar conditions, butylated hydroxytoluene (BHT) (at 50 ppm) only inhibited peroxides formation and TBARS by 25% and 20%, respectively, thus showing that seaweed extracts could be used as a potential substitute for synthetic antioxidants. In the same line, in a different food matrix, namely low-fat pork liver pâté, the incorporation of 500 mg/kg of a commercial antioxidant extract of *F. vesiculosus* was also shown to be as effective as 50 ppm of BHT at inhibiting the formation of primary and secondary oxidation products over 180 days under storage at 4 °C, as well as in the maintenance of the redness and yellowness which were lowered in the control samples [83]. On the other hand, the fortification of pork patties with *F. vesiculosus* 50% ethanol extracts (250–1000 mg/kg) showed low performances on samples oxidative stability, with modest inhibitory effects on TBARS, compared to the control samples, but very far from that exhibited by BHT. Additionally, regardless the good acceptability in the sensory analysis, the incorporation of these *F. vesiculosus* extracts failed to improve colour, surface discoloration or odour attributes [89]. Therefore, further studies are necessary to conclude whether extracts of this seaweed are suitable for the application as oxidation inhibitors for the long-term storage of meat products.

Further aiming lipid stabilization in dairies, O’Sullivan et al. [84,85] tested the incorporation of 0.25% and 0.5% (*w*/*w*) of 60% and 40% ethanol extracts from *F. vesiculosus* into milk and yogurt, respectively. Indeed, both products showed a significant reduction of lipid oxidation alongside with improvements on their shelf-life characteristics. However, neither were well accepted in the sensory analysis, even for the lower concentrations, as the panellists reported an unpleasant green/yellowish colour and a fishy taste.

Although the majority of the studies carried out with this seaweed species were focused on their antioxidant activity and capacity to enhance foods’ lipid stability, other authors have tried the incorporation of *F. vesiculosus* with different purposes. In a recent work, the incorporation of *F. vesiculosus* fucoidans into a new functional pasteurized apple beverage was found to be useful for controlling the growth of an undesirable microorganism, since strong bacteriostatic and bactericidal effects against *Listeria monocytogenes* and *Salmonella typhimuium* were observed in a dose-, time- and temperature-dependent manner [86]. On the other hand, Arufe et al. [87] studied the influence of the addition of different concentrations (2–8% *w*/*w*) of *F. vesiculosus* seaweed powder into wheat flour to the final rheological properties of the dough, such as the density and crumb texture. The authors found that for concentrations above 4%, the addition of *F. vesiculosus* powder caused the increase of the elongational dough viscosity and consequent decrease of its porosity, as well as the increase in the bread density, crumb firmness and appearance of a green colour. Therefore, 4% of *F. vesiculosus* powder would be the maximum amount that could be added to the bread without impairing its properties.

### 3.2. Himanthalia elongata

*H. elongata* has also been object of many studies comprising the development of seaweed-enriched foods, which, in addition to the improvement stability and/or shelf-life extension, also aimed to provide enhanced nutritional properties to the foods. In this field, many works reporting *H. elongata* fortified-foods were carried out on meat and meat-based products (Table 2). One of the most exploited attributes of this seaweed species is perhaps its wealthy mineral composition, which makes *H. elongata* a good candidate to be used as a salt replacer, contributing to the reduction of salt consumption and related health complications typical of western high-NaCl diets. It also increases the consumption of other elements, such as calcium potassium or iodine, which are usually lacking or below recommended levels in regular diets [4].

Many of these studies were carried out by the group of Jiménez-Colmenero et al., who have developed several meat products in which the content of sodium chloride was partially replaced by different species of edible seaweeds, including *H. elongata*. Among the seaweed-containing formulations, frankfurters, restructured meats and meat emulsions were shown to have at least 50 to 75% less NaCl than their conventional recipes [1,90,91,95,100,101]. Apart from the NaCl replacement, the fortification of frankfurters and meat emulsions with *H. elongata* also contributed to the increase of K content and subsequent reduction of the Na/K ratio from 3 to values below 1 (i.e., close to those recommended by WHO for maintaining a healthy cardiovascular condition). Additionally, the Ca, Mg and Mn contents in these two meat products increased to >1000%, >300% and >700%, respectively, compared with the conventional formulas, alongside with their water and fat binding properties [1,101]. Other effects resultant from *H. elongata* fortification in these matrices included the reduced cooking loss and increase in the Kramer shear force in restructured poultry meat [90]; increased water and oil retention in pork meat batter [92]; increased dietary fibre content in frankfurters [101]; and increased phenolic content and antioxidant activity in meat emulsions [91]. Overall, these products were well-accepted in the sensory analysis, with exception of frankfurters that were reported unpleasant mainly due to the increase of the dryness feeling and seaweed-like taste.

Cox and Abu-Ghannam [96] also reported that *H. elongata*-fortified beef patties (10–40% *w*/*w*) were very well accepted in the sensory analysis, particularly those with 40% of seaweed, getting even better scores than the control samples. This was mainly due to the improvements on the samples’ texture and overall mouthfeel, which resulted from the decrease in the cooking loss (associated to the incremented fibre content) and the increase in tenderness for more than 50%. Furthermore, a significant enhancement of the phenolic content and antioxidant activity (in a dose-dependent manner), as well as a lowered microbiological count and lipid oxidation before the chilling stage and after 30 days of storage, were observed in all patties containing seaweed. In fact, at the end of the experiment, the samples containing above 20% of *H. elongata*, showed no bacterial growth at all, as well as considerably low levels of the lipid oxidation marker.

In vivo studies on rat models revealed that the introduction of restructured pork meat enriched with 5% *H. elongata* (RPS) in the animals’ hypercholesterolemic diet significantly lowered the serum cholesterol levels that were augmented in the group under a non-RPS supplemented hypercholesterolemic diet. Moreover, a significant increase in SOD and GPx, alongside with a decrease of glutathione reductase (GR) expressions, were observed in both groups under hypercholesterolemic and regular RPS-supplemented diets, although increased glutathione reductase activity was also verified. Interestingly, the combined cholesterol and seaweed diet predisposed an increase in the expression of GR, SOD and liver cytochrome P450 7A1 (CYP7A1), i.e., a gene that encodes for the enzyme responsible for the elimination of cholesterol through the production of bile acids, but a decrease in the expression of CAT and GPx, suggesting a possible blocking effect of the hypercholesterolemic agent induced by seaweed incorporation [93]. In a similar study, rats under RPS-supplemented hypercholesterolemic diets, not only exhibited lower plasma cholesterol levels but also lower liver apoptosis markers, namely cellular cycle DNA, caspase-3 and cytochrome c [102]. Supporting these results, González-Torres et al. [94] confirmed that the administration of *H. elongata*-fortified restructured pork meat (at 5%) to rats under cholesterol-rich diets, partially blocked the hypercholesterolemic effect of the dietary pattern while changing the lipogenic/lipolytic enzyme expression (decreasing hormone-sensitive lipase and fatty acid synthase while increasing acetyl CoA carboxylase expressions compared with subjects under hypercholesterolemic diet) and reducing the wasting effect of hypercholesterolemia on adipose tissue in rats.

Apart from meat products, *H. elongata* powder has also been used to enrich breadsticks in order to enhance their nutritional properties. From the 10 formulations tested (with seaweed concentrations of 2.63 to 17.07% *w*/*w*), the highest was reported to have the most significant influence on the chemical properties of breadsticks. Furthermore, this sample also had higher levels of total dietary fibre, while the total phenolic content and antiradical activity were maximized at 138.25 mg GAE/100 g dry basis and 61.01%, respectively, maintaining an acceptable edible texture and colour of the samples. Therefore, since no significant difference was seen between the control and seaweed enriched breadsticks in terms of sensory analysis, this product could have great acceptability, especially to non-seaweed consumers [97]. The augmented phenolic content as well as the enhanced antioxidant activity were also described on functional breads developed with 8% of *H. elongata* flour [98]. On the other hand, an attempt to supplement yogurt and quark with dehydrated *H. elongata* (0.25–1% *w*/*w*) turned out to negatively affect almost all the sensory parameters analysed, which makes this seaweed not very suitable for application in these two dairies, at least in these conditions [99].

### 3.3. Undaria pinnatifida

Similar to *H. elongata*, the applications of *U. pinnatifida* as functional ingredients have mostly been reported in meat and meat-derived products (Table 3). For instance, the incorporation of *U. pinnatifida* (1–4%) into pork beef patties increased their ash content as well as their juiciness due to the lower cooking losses compared to the control [103]. In a similar approach, the reformulation of low-salt (0.5%) and low-fat (<10%) beef patties by the addition of 3% of *U. pinnatifida* and partial or total replacement of pork backfat with olive oil-in-water emulsion, significantly affected the frozen storage characteristics of the products. This presented enhancements in terms of technological, sensory and nutritional properties, as well as improvements in their physiological benefits. These reformulated patties demonstrated less thawing and cooking losses, and were texturally softer than the samples without seaweed, most likely due to the microstructural changes caused by the formation of alginate chains.

Moreover, the incorporation of *U. pinnatifida* in the patties’ formulation did not hamper their lipid oxidation or microbiological counts, and although the content of Na and K were twice as high as the control samples, the Na/K ratio were still close to 1. Likewise, magnesium and calcium levels were higher in seaweed-fortified samples, corresponding three and six-fold, respectively, to those of the conventional recipe. Interestingly, although a different flavour was pointed out in the sensory analysis, panellists generally described the reformulated patties to be more pleasant and palatable than the control [104]. This reformulation with *U. pinnatifida* also resulted in significant improvements in several parameters on cooked patties, namely in the binding properties and retention values of moisture, ash and particularly fat and fatty acids, the latter parameter being usually the most affected by the cooking process. This means that the incorporation of this seaweed in the patties greatly interfere with the fat and energy content of these food matrices, as well as their fatty acids profile [105]. Identical results were reported on low-salt gel/emulsion meat systems added with 2.5–5% of *U. pinnatifida*, which exhibited better firmness and chewiness due to improvements of the water and fat-biding properties [1]. The incorporation of 5.6% of this species in such systems was also reported to contribute to the increment of the products’ phenolic content and antioxidant properties, as well as to improve their mineral profile, increasing the K, Mg, Ca and Mn contents while decreasing the Na content, thus consequently reducing the Na/K ratio from 3.5 in the control samples, to approximately 1. Contrastingly, despite the potential beneficial health effects, increasing the algae was considered a non-satisfactory strategy to achieve healthier lipid meat formulations, since it could affect the food’s sensory properties and their lipid content was very low [91]. In turn, Sasaki et al. [106] observed that the addition of 200 mg/kg fucoxanthin extract from *U. pinnatifida* to raw ground chicken breast meat did not prevent the lipid oxidation during their freeze storage period (1 or 6 days). However, it did inhibit TBARS formation of cooked samples stored under the same conditions and improved the products’ overall appearance, indicating that fucoxanthin could prevent the oxidation in these products and effectively extend their shelf-life.

Apart from the nutritional stability of the foods, the incorporation of *U. pinnatifida* into foods have also been demonstrated to have great beneficial effects in distinct parameters with impact in the cardiovascular system. According to Moreira et al. [108], the administration of *U. pinnatifida*-fortified restructured pork meat to Wistar rats under a cholesterol-rich diet, not only caused the lowering of the plasma redox index by increasing total and reduced glutathione together with the GR and SOD activity, but also contributed to the decrease of the caspase-3 activity and therefore, hypercholesterolemic-induced apoptotic response of their hepatocytes [102].

Only few studies have focused the use of *U. pinnatifida* in products other than meat. Nevertheless, Prabhasankar et al. [107] reported significantly higher phenolic content and antioxidant activity in the aqueous extracts of uncooked pasta containing different concentrations of *U. pinnatifida* (5–30% *w*/*w*) compared to the controls. Although the cooking process caused a loss in these two parameters, they were still significantly higher on seaweed-added pasta compared to the values observed in the conventional pasta. Importantly, the heat processes involved in pasta preparation and cooking did not damaged fucoxanthin. The seaweed incorporation also contributed to the improvement of the pasta amino acid and fatty acid profiles, as well as the increase of bioactive compounds. The pasta incorporated with 10% seaweed, which demonstrated the highest radical scavenging activities, was also the most well accepted in the sensory analysis. The augmented phenolic content and antioxidant activity were also described on functional breads developed with 8% of *U. pinnatifida* flour, although other seaweeds, such as *H. elongata* exhibited better results [98].

The incorporation of *U. pinnatifida,* up to 15% in cottage cheeses, was reported to cause a dose-dependent increment of their Ca, Fe and Mg. However, the textural quality was best for cheeses containing 9% of seaweed [109]. On the other hand, Nuñez and Picon [99] found that, among the 5 different seaweeds used to incorporate in yogurts and quark cheese, dehydrated *U. pinnatifida* at 0.5% (*w*/*w*) was the formulation that showed the highest seaweed flavour and the lowest flavour quality in both dairies, worsening almost all of their sensory aspects and making this seaweed unattractive for application in such dairies. To overcome this disadvantage, it would be interesting to explore alternative approaches, such as the application of seaweed in flavoured dairies, the application of algae extracts instead of whole algae or the encapsulation of algae or extracts thereof, in order to assess whether these or other strategies could mask the negative impacts that *U. pinnatifida* has on the sensory aspects of these dairies.

### 3.4. Ascophyllum Nodosum

Although *A. nodosum* has not been much studied as a functional ingredient for incorporation in foods, some authors have reported promising results in this field (Table 4). For instance, Dierick et al. [110] found that, feeding pigs with 20 g of *A. nodosum*/kg of feed over 21 days caused the levels of iodine in muscle and internal organs to increase 2.7 and 6.8 times, respectively, compared to the pigs fed under a regular diet. This could be a viable approach to increase the daily intake of this mineral which is usually deficient in several European countries [4]. Alternatively, *A. nodosum* extracts applied to low-fat pork liver pâtés (500 mg/kg) was described to increase the protein content by approximately 4% compared to the control samples, without interfering with the chemical composition or microbial characteristics of the samples, throughout 180 days of storage at 4 °C. Furthermore, at the end of the experiment, the oxidative parameters on seaweed-added samples were comparable to those of BHT-added samples, both showing a similar degree of protection against oxidation as well as a significant reduction of volatile compounds after storage [83].

On another perspective, *A. nodosum* extracts have proven to be effective in the inhibition of lipid oxidation and the improvement of antioxidant activity in dairies. Indeed, the incorporation of either aqueous or 80% ethanol extracts (0.25% and 0.5%) of this species in milk significantly decreased the TBARS formation and increased the radical scavenging and ferrous-ion-chelating activities either before or after in vitro digestion. However, this did not affect the cellular antioxidant activity or protect against DNA damage in human colon adenocarcinoma Caco-2 cells, suggesting that the fortification with *A. nodosum* extracts could improve certain milk qualities and shelf-life characteristics, but not provide significant biological activity. Interestingly, despite fortified-milk with aqueous extract had good acceptability in the sensory analysis, those formulated with 80% ethanol extract was pointed to have a fishy taste and off flavour, thus having low acceptability by the panellists. Nevertheless, this issue could potentially be addressed by using food flavourings or through micro-encapsulation to camouflage the undesirable flavours [84]. A new set of studies on fortified yogurts with the same *A. nodosum* extracts also revealed the increment of the radical scavenging activity before and after in vitro digestion, which was shown not to affect parameters, such as the product’s acidity, microbiology or whey separation. However, as previously stated, the biological activity on cellular models was absent and the sensorial analysis was positive for *A. nodosum* aqueous extracts but not for the 80% ethanol extracts [85]. On another approach, Hall et al. [111] reported that the addition of *A. nodosum* (1–4%) in bread significantly reduced the energy intake after a test meal in a single blind cross trial. Moreover, the same was verified after 24 h of seaweed-enriched bread consumption and no differences were observed in blood glucose and cholesterol levels. The authors highlighted, however, the need of a long-term interventional study to establish the real potential of *A. nodosum*-enriched bread energy intake, in addition to the metabolism of glucose and lipids.

### 3.5. *Laminaria* sp.

*Laminaria* is one of the most economically important algae genus since it comprises 31 species, being most widely exploited worldwide as raw materials for alginates production [112]. On the other hand, the studies focusing the use of these seaweeds as functional ingredients in foods are quite limited (Table 5). Nevertheless, due to their high content in iodine, some authors have investigated the use of *Laminaria* sp. as animal feed aiming to increase the iodine content in their muscle before slaughter. Indeed, the work carried out by Schmid et al. [113] demonstrated that feeding chars (*Salvelinus* sp.) with *L. digitata*-fortified fish meal (0.8%) over nine months, contributed to an increase of their total iodine content in approximately four times the levels found in the control fishes. Similar observations were described in other species, such as gilthead seabream (*Sparus aurata*) and rainbow trout, which revealed an increased iodine content in their fillets after *L. digitata* was introduced in their meals as well [114,115]. An identical experiment carried out with pigs also revealed that the supplementation of *L. digitata* in the animal’s feed over 3 months resulted in an accumulation of 45% more I in muscle tissue and up to 213% in other internal organs compared to the pigs under a normal diet [116]. In a different approach, four group of pigs were assigned to different diets 35 days pre-slaughter in order to test whether alterations of their diets would affect bacterial count, lipid peroxidation and total antioxidant capacity of fresh meat during storage. Interestingly, the meat excised from the group fed with the *Laminaria* sp.-supplemented diet exhibited the best overall results, showing the highest antioxidant activity, the lowest lipid peroxidation and microbial counts, suggesting that feeding the animals with seaweeds might have a significant impact on the quality and shelf-life of their meat [117].

Alternatively, Moroney et al. [118] tested whether the incorporation of different concentrations (0.01%, 0.1% and 0.5% *w*/*w*) of *L. digitata* extract, containing laminaran and fucoidan in chopped pork patties would affect their quality and shelf-life period. The results showed that the surface redness of fortified raw patties, upon 14 days under modified atmosphere packages at 4 °C, decreased compared to the control samples, which led to a slight decrease of their quality parameters. Fortification with the extract at 0.5% caused a notable reduction of lipid oxidation in the cooked samples, but the formulated product was not very well accepted in the sensory analysis. A similar work was later conducted with fresh and cooked pork homogenates and commercial horse heart oxymyoglobin incorporated with *L. digitata*-extracted fucoidan, laminaran and a mixture of both. Although fucoidan showed the strongest radical scavenging activity, cooking and digestion of the samples caused a significant decrease of the antioxidant potential in the samples added with this fibre, which could possibly be attributed to its more acidic nature. Interestingly, despite this, polysaccharide was found to reduce lipid oxidation and also was responsible for catalysing the oxidation of oxymyoglobin. Notably, when the digested samples containing the mixture of laminaran and fucoidan were evaluated for their bioaccessibility in a Caco-2 cell model, a decrease in radical scavenging activity of 44.2% and 36.6% was observed after 4 and 20 h of incubation, indicating a theoretical uptake of these polysaccharides. These results highlight the potential use of seaweed extracts as functional ingredients in pork with the advantage of possibly improving the human antioxidant defences [42].

In addition to *L. digitata*, other species of this genus have been reported for their positive effects as functional ingredients in foods. This is the case of *Laminaria japonica*, which was incorporated (1–4% *w*/*w*) in breakfast sausages contributing to a significant dose-dependent increase of their ash content, as well as to the improvements on the emulsion stability and textural parameters such as hardness, gumminess and chewiness. Moreover, the seaweed addition lowered samples’ pH, lightness, redness and yellowness, and lowered cooking and water losses, particularly in samples added with 4%. Nevertheless, despite the higher benefits that were observed for higher seaweed powder concentrations, the sensory evaluations determined that the 1% *L. japonica* sausage had the highest overall acceptability [119]. In addition, the incorporation of *L. japonica* in chicken or pork patties was inclusively demonstrated to have positive effects in the post-plasma glucose and lipids profiles in borderline-hyperlipidaemic adults voluntaries. The consumption of fortified-patties with 2.25 g of this species not only lowered the increased post-prandial serum glucose levels compared to the control group, but also the total cholesterol and low density lipoprotein concentrations, while maintaining the same levels of high density lipoprotein [120].

In an alternative to meat products, a new probiotic yogurt containing different concentrations of *Laminaria* sp. was developed with the aim of increasing its iodine content. Indeed, contrarily to the conventional yogurt, the fortified formulation contained not only high levels of I (average of 570 µg I/100 g), but also considerably incremented amounts of Ca, K, Na, Mg, and Fe [121], overall improving their mineral profile.

## 4. Future Considerations

Previous studies focusing brown seaweed-fortified products have demonstrated that in general, macroalgae can improve the nutritional value of the food products, either by incrementing levels of dietary fibres and/or minerals or their lipidic profiles. Thus, fortified foods with seaweed and/or seaweed extracts come out as possible nutritional alternatives to the original formulations. Moreover, the reported information seems to be solid regarding the fact that the fortification of foods with brown seaweeds and/or their extracts in general have positive impacts on both their oxidative stability and microbial inhibition effects. However, discrepant results are reported regarding the technological properties of the fortified products, namely on the stability of the food’s structure. Hence, there is the need to guarantee the compatibility of seaweeds and the overall food matrix, which is not only a result of the seaweed itself, but of the combination of the seaweed with the proper ingredients. In addition, the incorporation of seaweeds in foods frequently comprises their sensorial attributes due to colour changes and the appearance of off-flavours. Nevertheless, some strategies such as fermentation, enzymatic processing or encapsulation of seaweeds or their extracts have already shown interesting effects at cloaking seaweeds’ negative sensorial characteristics while maintaining their nutritional properties and stability of bioactive components [122,123,124]. Nevertheless, further research in this field is necessary to understand if the cost-benefit of the application of such techniques is viable on a larger scale.

Being a crucial factor in nutrition, the bioavailability of relevant nutrients and/or phytochemicals is another critical issue that will require much attention in the following years. This is highly dependent on food components and on individual gastrointestinal conditions. Alginic acid, fucoidans and laminarans are considered dietary fibres, meaning that they may be fermented by colon microflora, therefore surviving the majority of the digestion [125]. The rest of the compounds seem, however, to be absorbed at earlier stages. For instance, in vitro studies suggested that dietary fucoxanthin is metabolized to fucoxanthinol and amarouciaxanthin A [126,127]. In fact, daily administered dietary fucoxanthin (*L. japonica* and *U. pinnatifida* origin) was shown to accumulate as amarouciaxanthin A and fucoxanthinol in several mice tissues [128,129]. In humans, however, the plasma concentrations of fucoxanthin metabolites before and after 1-week dietary interventions with *U. pinnatifida* were shown to be either low (fucoxanthinol) or non-existent (fucoxanthin and amarouciaxanthin A), although a higher subject group would be required in order to confirm these results [130].

As for phlorotannins, to the authors knowledge, only one bioavailability study was made using this particular compound from any of the seaweeds of interest to this review. Recently, in a work developed by Corona et al. (2016), a food-grade phlorotannin extract from *A. nodosum* was submitted to in vitro and in vivo assays, the latter involving the oral administration of a 100 mg capsule with the same extract [57]. The in vitro digestion and fermentation allowed for the identification of 11 compounds including hydroxytrifuhalol A, a C-O-C dimer of phloroglucinol, diphlorethol/difucol and 7-hydroxyeckol, some of which were also detected in the urine and plasma of human participants, thus confirming their absorption into the blood circulation. Moreover, although brown seaweeds are considered a great source of iodine, there is limited information regarding its bioavailability. Domínguez-Gonzaléz et al. [131] found out that despite the high in vitro bioaccessibility of iodine from *U. pinnatifida* and *S. japonica*, only a small percentage was bioavailable using dialysis membranes and an even lower in a biological system model consisting of two major cell types present in the intestine. Nevertheless, more favourable results were demonstrated when iodine-insufficient women were supplemented with encapsulated *A. nodosum*, since one third of the ingested iodine was found to be bioavailable [132].

Hence, it is clear that, not only is there a lack of information regarding the bioavailability of nutrients/phycochemicals in seaweeds and seaweeds-fortified foods in general, but also the relationship between seaweed-fortified products and their potential functionality remains almost unexploited. Indeed, evidences of biological effects of seaweeds-fortified products were barely proven in cellular models and even more rarely in in vivo trials and hence, must still be made to assure the conformity of the results. According to Plaza et al. [133], the principal guideline to follow in the design of a new functional food is to increase as much as possible the benefit/risk ratio, by increasing the benefit to the maximum and reducing the risk to the minimum, considering toxicity studies, for example. Increasing the benefit implies looking for a physiological wide effect, assuring the existing bioavailability and that the mentioned bioavailability is going to be kept along all the useful life of the food. Therefore, since the in vivo biological activity of phycochemicals depends on their bioavailability, in the future, it would be interesting to further access how important properties claimed for brown algae can transpose to human beings through seaweed fortified products. In order to reduce the risk, it is necessary to carry out toxicity studies, to use the functional ingredient in minimal effective doses and to use as functional ingredients, the products naturally found in foods or natural sources.

## 5. Conclusions

In conclusion, seaweeds are very valuable food sources with reportedly high nutritional value and high in bioactive compounds. In this regard, brown macroalgae are particularly known to accumulate specific metabolites, such as the polysaccharides alginic acids, laminarans and fucoidans, the phenolic compounds phlorotannins, the carotenoid fucoxanthin and exceptionally high levels of iodine with simultaneous low Na/K ratios, which overall confer them great potential to be used as functional ingredients. Thus far, most of the recent studies’ main objectives focusing on testing the incorporation of macroalgae in foods, namely those considered as safe for consumption in Europe i.e., *F. vesiculosus, F. serratus, H. elongata*, *U. pinnatifida*, *A. nodosum*, *L. digitata, L. saccharina, L. japonica* and *A. esculenta*, were the increment of the product’s shelf-life and the sensory properties culminating in the potential commercialization. From an economic point of view, this is rather important, but in terms of the benefits they could bring to the consumer, there is a lot of work to be done. Therefore, in order to effectively exploit this very promising raw material, the focus should be on their bioavailability, especially in humans.

## Figures and Tables

**Table 1:** Selected studies reporting the effects of the incorporation of *F. vesiculosus* or isolates as ingredients in different food matrices.

Functional Food	Functional Ingredient	Results	Ref.
Fish cakes	*F. vesiculosus* extracts: 100% H_2_O, 80% EtOH	No off-flavours and lower rancid odour and flavour None of the extracts had influence on lipid oxidation nor quality of the products	[73]
Cod muscle and protein isolates	*F. vesiculosus* 80% EtOH extract and further fractions (EtOAc + Sephadex LH-20)	↓ Lipid oxidation in both fish muscle and protein isolates 300 mg/kg of the oligomeric phlorotannin fractions exhibited an effect comparable to that of 100 mg/kg propyl gallate	[75]
Cod mince	EtOAc fraction of *F. vesiculosus* 80% EtOH extract	↓ Lipid oxidation in fish muscle	[76]
Cod protein hydrolysates	EtOAc fraction of *F. vesiculosus* 80% EtOH extract	↓ Lipid hydroperoxide and TBARS formation during protein hydrolyzation ↑ Antioxidant activity of the final protein hydrolysates	[78]
Cod protein hydrolysates	EtOAc fraction of *F. vesiculosus* 80% EtOH extract	↓ Lipid oxidation during protein hydrolysates freeze drying ↑ Antioxidant activity of the final protein hydrolysates Improved sensorial aspects (bitter, soap, fish oil and rancidity taste)	[77]
Minced horse mackerel	*F. vesiculosus* antioxidant dietary fibre	↓ Lipid oxidation during 5 months of storage at −20 °C ↓ Total dripping after thawing and cooking after up to 3 months of frozen storage Improved fish mince flavour	[74]
Granola bars enriched with fish oil emulsion	*F. vesiculosus* 100% H_2_O, 70% acetone and 80% EtOH extracts	↓ Oxidation products after storage ↓ Iron-lipid interactions Acetone and EtOH extracts provided additional lipid oxidation protection ↑ Phenolic content, radical scavenging activity and interfacial affinity of phenolic compounds Possible tocopherol regeneration	[79]
Granola bars enriched with fish oil emulsion	*F. vesiculosus* 100% H_2_O, 70% acetone and 80% EtOH extracts	↓ Lipid oxidation during storage ↑ Effectiveness for lower concentrations of EtOH and acetone extracts ↑ Phenolic content, radical scavenging activity and interfacial affinity of phenolic compounds Possible tocopherol regeneration	[80]
Fish-oil-enriched milk and mayonnaise	*F. vesiculosus:* EtOAc fraction from an 80% EtOH extract, 100% H_2_O	↑ Lipid stability and ↓ oxidation of EPA and DHA and subsequent secondary degradation products in both foods—H_2_O extract at 2.0 g/100 g exerted higher inhibitory effects on mayonnaise’s peroxide formation.	[81]
Fish-oil-enriched mayonnaise	*F. vesiculosus* 100% H_2_O, 70% acetone, and 80% EtOH extracts	Dose-dependent inhibition of lipid oxidation exhibited by EtOH and acetone extracts H_2_O extract increased peroxide formation	[82]
Pork liver pâté	*F. vesiculosus* commercial extract	Decrease in lightness values after storage Redness and yellowness maintained after storage Protection against oxidation comparable to BHT samples ↓ Total volatile compounds	[83]
Pork patties	*F. vesiculosus* 50% EtOH extracts	↓ TBARS slightly Did not improve colour, surface discoloration or odour attributes No significant differences between seaweed and control samples in sensory analysis	
Milk	*F. vesiculosus* 60% EtOH extracts	↑ Milk lipid stability and shelf-life characteristics Appearance of greenish colour and fishy taste Overall sensory attributes were worsened	[84]
Yoghurts	*F. vesiculosus* 60% EtOH extracts	No influence on chemical and microbiological characteristics ↑ Yogurts lipid stability and shelf-life characteristics Overall sensory attributes were worsened	[85]
Pasteurized apple beverage	*F. vesiculosus* fucoidan extract	Dose-, time- and temperature-dependent bacteriostatic and bactericidal effects against *L. monocytogenes* and *S. typhimurium* *S. typhimurium* showed higher sensitivity to the extract	[86]
Bread	*F. vesiculosus* powder	↑ Dough viscosity and wheat dough consistency ↓ Porosity ↑ Density, crumb firmness and green colour of crust 4% seaweed powder was considered optimal	[87]

↑: increased; ↓: decreased; BHT: 2,6-di-*tert*-butyl-4-methylphenol; DHA: docosahexaenoic acid; EPA: eicosapentanoic acid; EtOAc: ethyl acetate; EtOH: ethanol; TBARS: Thiobarbituric acid reactive substances.

**Table 2:** Selected studies reporting the effects of the incorporation of *H. elongata* or isolates as ingredients in different food matrices.

Functional Food	Functional Ingredient	Results	Ref.
Poultry steaks	3% dry matter *H. elongata*	↑ Purge loss slightly ↓ Cooking loss ↑ Levels of total viable counts, lactic acid bacteria, tyramine and spermidine No important changes observed during chilled storage Positive overall acceptance by a sensory panel	[90]
Pork gel/emulsion systems	2.5% and 5% dry matter *H. elongata*	↑ Water and fat binding properties ↑ Hardness and chewiness of cooked products ↓ Springiness and cohesiveness	[1]
Low-salt pork emulsion systems	5.6% dry matter *H. elongata*	↑ Content of n-3 PUFA ↓ n-6/n-3 PUFA ratio ↓ Thrombogenic index ↑ Concentrations of K, Ca, Mg and Mn	[91]
Pork meat batter	3.4% powder *H. elongata*	↑ Water/oil retention capacity, hardness and elastic modulus. Thermal denaturation of protein fraction was prevented by seaweed alginates Nutritional enhancement	[92]
Restructured meat	5% powder *H. elongata*	Effects in rats: ↓ Total cholesterol ↑ CYP7A1, GPx, SOD, GR expression ↓ CAT expression	[93]
Restructured meat	5% powder *H. elongata*	↓ HSL and FAS and ↑ ACC (*p* < 0.05) expression on rats fed with seaweed fortified meat comparing with rats under hypercholesterolemic diet	[94]
Frankfurters	3.3% *H. elongata* powder	↑ Cooking loss ↓ Emulsion stability Combination of ingredients provided healthier meat products with lower fat and salt contents Worsened physicochemical and sensory characteristics	[95]
Beef patties	10–40% (*w*/*w*) *H. elongata*	↓ Cooking loss ↑ Tenderness, dietary fibre levels, TPC and antioxidant activity ↓ Microbiological counts and lipid oxidation Patties with 40% seaweed had the highest overall acceptability	[96]
Bread sticks	2.93–17.07% *H. elongata* powder	Highest concentration had higher phycochemical constituents, acceptable edible texture and overall colour	[97]
Bread	8% (*w*/*w*) *H. elongata*	↑ TPC ↑ Antioxidant activity in DPPH^•^, ORAC and TEAC	[98]
Yoghurt and Quark	0.25–1% dehydrated *H. elongata*	Alterations in all yoghurt attributes except for buttery odour, and acid and salty flavours Alterations in all quark attributes except yogurt odour, acid flavour and sweet flavour. Sensory characteristics worsened	[99]

↑: increased; ↓: decreased; ACC: acetyl CoA carboxylase; CAT: Catalase; CYP7A1: liver cytochrome P450 7A1; DPPH^•^: 2,2-diphenyl-1-picrylhydrazyl radical; FAS: fatty acid synthase; GPx: Glutathione peroxidase; GR: Glutathione reductase; HSL: hormone-sensitive lipase; ORAC: oxygen radical absorbance capacity; PUFA: polyunsaturated fatty acids; SOD: superoxide dismutase; TEAC: trolox equivalent antioxidant capacity; TPC: Total phenolic content.

**Table 3:** Selected studies reporting the effects of the incorporation of *U. pinnatifida* or isolates as ingredients in different food matrices.

Functional Food	Functional Ingredient	Results	Ref.
Beef patties	3% dry matter *U. pinnatifida*	↑ Binding properties and cooking retention values of, fat, fatty acids and ash Replacement of animal fat with olive-in-water emulsion and/or seaweed was reportedly healthier. ↓ Thawing and↑ softer texture Changes on the microstructure due to formation of alginate chains Overall acceptable products and fit for consumption	[104,105]
Chicken breast	200 mg/kg *U. pinnatifida*	↑ Redness and yellowness ↓ Lipid oxidation in chilling storage and after cooking Overall appearance and shelf-life were enhanced	[106]
Pork gel/emulsion systems	2.5% and 5% dry matter *U. pinnatifida*	↑ Water and fat binding properties ↑ Hardness and chewiness of cooked products ↓ Springiness and cohesiveness	[1]
Low-salt pork emulsion systems	5.6% dry matter *U. pinnatifida*	↑ Content of n-3 PUFA ↓ n-6/n-3 PUFA ratio ↑ Concentrations of K, Ca, Mg and Mn ↑ Antioxidant capacity	[91]
Pasta	100:0, 95:5, 90:10, 80:20 and 70:30 (semolina/*U. pinnatifida*; *w*/*w*)	10% *U. pinnatifida* was the most acceptable ↑ Amino acid, fatty acid profile and nutritional value of the product Fucoxanthin was not affected by pasta making and cooking step	[107]
Yoghurt and Quark	0.25–1% dehydrated *U. pinnatifida*	↑ Seaweed flavour with ↓ flavour quality for 0.5% seaweed Alterations in all yoghurt attributes except for buttery odour, and acid and salty flavours Alterations in all quark attributes except yogurt odour, and acid and sweet flavours. Sensory characteristics worsened	[99]
Bread	8% (w:w) *U. pinnatifida*:wheat flour	↑ TPC, ↑ Antioxidant activity in DPPH^•^, ORAC and TEAC	[98]

↑: increased; ↓: decreased; DPPH^•^: 2,2-diphenyl-1-picrylhydrazyl radical; ORAC: oxygen radical absorbance capacity; PUFA: polyunsaturated fatty acids; TEAC: trolox equivalent antioxidant capacity; TPC: Total phenolic content.

**Table 4:** Selected studies reporting the effects of the incorporation of *A. nodosum* or isolates as ingredients in different food matrices.

Functional Food	Functional Ingredient	Results	Ref.
Pork	20 g *A. nodosum* /kg feed	↑ I content in piglet’s muscles and internal organs	[110]
Pork liver paté	*A. nodosum* extract at 500 mg/kg	↑ Protein content ↑ Redness and yellowness after storage Degree of protection against oxidation comparable to BHT samples ↓ Total volatile compounds	[83]
Milk	*A. nodosum* (100% H_2_O and 80% EtOH) extracts (0.25 and 0.5 (*w*/*w*))	↓ TBARS formation ↑ Radical scavenging and ferrous-ion-chelating activities before and after digestion Supplementation on Caco-2 cells did not affect cellular antioxidant status EtOH extracts had greenish colour and overall sensory attributes were worsened	[84]
Yoghurts	*A. nodosum* (100% H_2_O and 80% EtOH) extracts (0.25 and 0.5 (*w*/*w*))	No influence on chemical characteristics Yoghurts had antioxidant activity before and after digestion Supplementation on Caco-2 cells did not affect cellular antioxidant status Overall sensory attributes were worsened	[85]
Bread	1–4% *A. nodosum* per 400 g loaf	All samples sensorially accepted ↓ Energy intake after 4 h Glucose and cholesterol blood levels not affected	[111]

↑: increased; ↓: decreased; BHT: butylated hydroxytoluene; EtOH: ethanol; TBARS: thiobarbituric acid reactive substances.

**Table 5:** Selected studies reporting the effects of the incorporation of *Laminaria* sp. or isolates as ingredients in different food matrices.

Functional Food	Functional Ingredient	Results	Ref.
Chars	*Laminaria digitata* (0.8% in fish meal)	↑ 4 times the I content in fish muscle	[113]
Gilthead seabream	*Laminaria digitata* (10% in fish meal)	↑ I content in fish fillets	[114]
Rainbow trout	*Laminaria digitata* (0.65% in fish meal)	↑ I content in fish fillets	[115]
Pork	*Laminaria digitata* (1.16 and 1.86 g/kg feed)	↑ I content in pigs’ muscles by 45% and internal organs by 213%	[116]
Pork	*Laminaria digitata* (5.32 kg/t feed)	↑ Antioxidant activity ↓ Lipid oxidations ↓ Microbial counts	[117]
Pork patties	0.01%, 0.1% and 0.5% (*w*/*w*) of 9.3% laminarin and 7.8% fucoidan from *L. digitata*	↑ Lipid antioxidant activity for L/F extract (0.5%) No effect in colour, lipid oxidation, texture or sensorial acceptance when adding L/F extract	[118]
Pork homogenates	3 and 6 mg/mL of laminaran, fucoidan and both from *L. digitata*	L had no antioxidant activity The L/F extract had higher antioxidant activity than F, after cooking and post digestion of minced pork. DPPH^•^ antioxidant activity lower in Caco-2 cell model with L/F extracts Seaweed extracts containing F had higher antioxidant activity of the functional cooked meat products.	[42]
Sausages	1–4% *L. japonica* powder	No changes in moisture, protein, and fat contents ↓ Lightness and redness values ↓ Cooking loss ↑ Emulsion stability, hardness, gumminess, and chewiness 1% seaweed had highest overall acceptability	[119]
Pork/chicken patties	*Laminaria japonica* (replacement od 2.25 g of pork/chicken for an equal amount of seaweed)	↓ Increased in postprandial glucose blood levels; ↓ TC and LDL-C	[120]
Yoghurt	*Laminaria* spp. (0.2% or 0.5% *w*/*w*)	↑ I, Ca, K, Na, Mg, and Fe	[121]

↑: increased; ↓: decreased; DPPH^•^: 2,2-diphenyl-1-picrylhydrazyl radical; F: fucoidan; L: Laminarin; LDL-C: low density lipoprotein; L/F: Laminarin and Fucoidan; TC: total cholesterol.
